# Cystatin B deficiency results in sustained histone H3 tail cleavage in postnatal mouse brain mediated by increased chromatin-associated cathepsin L activity

**DOI:** 10.3389/fnmol.2022.1069122

**Published:** 2022-11-30

**Authors:** Eduard Daura, Saara Tegelberg, Paula Hakala, Anna-Elina Lehesjoki, Tarja Joensuu

**Affiliations:** ^1^Folkhälsan Research Center, Helsinki, Finland; ^2^Faculty of Medicine, Medicum, University of Helsinki, Helsinki, Finland

**Keywords:** cystatin B, cathepsin L, EPM1, brain development, histone cleavage, H3cs1, histone H3, senescence

## Abstract

Cystatin B (CSTB) is a cysteine cathepsin inhibitor whose biallelic loss-of-function mutations in human result in defects in brain development and in neurodegeneration. The physiological function of CSTB is largely unknown, and the mechanisms underlying the human brain diseases remain poorly understood. We previously showed that CSTB modulates the proteolysis of the N-terminal tail of histone H3 (H3cs1) during *in vitro* neurogenesis. Here we investigated the significance of this mechanism in postnatal mouse brain. Spatiotemporal analysis of H3cs1 intensity showed that while H3cs1 in wild-type (*wt*) mice was found at varying levels during the first postnatal month, it was virtually absent in adult brain. We further showed that the high level of H3cs1 coincides with chromatin association of *de novo* synthesized cathepsin L suggesting a role for nuclear cathepsin L in brain development and maturation. On the contrary, the brains of *Cstb*^–/–^ mice showed sustained H3cs1 proteolysis to adulthood with increased chromatin-associated cathepsin L activity, implying that CSTB regulates chromatin-associated cathepsin L activity in the postnatal mouse brain. As H3 tail proteolysis has been linked to cellular senescence *in vitro*, we explored the presence of several cellular senescence markers in the maturing *Cstb*^–/–^ cerebellum, where we see increased levels of H3cs1. While several markers showed alterations in *Cstb*^–/–^ mice, the results remained inconclusive regarding the association of deficient CSTB function with H3cs1-induced senescence. Together, we identify a molecular role for CSTB in brain with implications for brain development and disease.

## Introduction

The cystatin B (CSTB) protein has been characterized in detail as an endogenous inhibitor of lysosomal cysteine cathepsins (Stubbs et al., 1990; Turk and Bode, 1991). Biallelic partial loss-of-function mutations in the *CSTB* gene (Pennacchio et al., 1996) underlie Unverricht-Lundborg disease (progressive myoclonus epilepsy type 1, EPM1; OMIM 254800), a neurodegenerative disorder that usually manifests during late childhood or early adolescence (Lehesjoki and Kälviäinen, 2020). The key clinical features include treatment-resistant disabling myoclonus, tonic-clonic seizures and ataxia with widespread progressive brain degeneration in imaging studies. The overall cognitive functions of EPM1 patients are mostly within normal range (Äikiä et al., 2021). In contrast, biallelic total loss-of-function *CSTB* mutations cause a neonatal-onset developmental encephalopathy with progressive microcephaly (Mancini et al., 2016; O’Brien et al., 2017). The severity of the phenotype thus correlates with the degree of CSTB expression in cells.

A *Cstb* knockout mouse (*Cstb*^–/–^) mimics the timeline and clinical features of human EPM1, including myoclonus with onset by postnatal day 30 (P30) and appearance of ataxia by 6 months of age (Pennacchio et al., 1998). Previous studies have shown that the presymptomatic phase in *Cstb*^–/–^ mice is characterized by microglial activation and dysfunction, reduced GABAergic inhibition, and aberrant transcriptional signatures associated with early synaptic changes and inflammation, and alterations in the synaptic mitochondrial proteome (Tegelberg et al., 2012; Joensuu et al., 2014; Okuneva et al., 2015; Gorski et al., 2020). The symptomatic onset is marked by the appearance of reactive astroglia and by the acceleration of neurodegenerative processes that culminates in pronounced brain atrophy affecting both gray and white matter (Buzzi et al., 2012; Tegelberg et al., 2012; Manninen et al., 2013, 2014).

The progressive brain degeneration observed in EPM1 patients and in *Cstb*^–/–^ mice, and the severe neonatal-onset phenotype in patients with biallelic *CSTB* null mutations indicate that CSTB is critically involved in brain development and homeostasis. However, its physiological function remains elusive and, consequently, the mechanisms linking CSTB deficiency to neurodegeneration are not understood.

In a human glioblastoma cell line, CSTB co-immunoprecipitated with histones and with the cysteine protease cathepsin L (Čeru et al., 2010). In a model of *in vitro* neurogenesis, we previously reported that CSTB regulates the cysteine cathepsin mediated proteolytic processing of the N-terminal tail of histone H3 between amino acids Ala21 and Thr22 (Daura et al., 2021), an evolutionarily conserved histone modification commonly known as H3 cleavage site 1 (H3cs1) (Duncan et al., 2008; Santos-Rosa et al., 2009). In mammalian cells and tissues, upregulation of H3cs1 has been linked to chromatin reprogramming events such as cellular differentiation and senescence, through permanent removal of post-translational modifications from histone proteins (Duarte et al., 2014; Azad et al., 2018; Cheung et al., 2021; Ferrari et al., 2021).

To gain insight into the mechanisms associated with CSTB deficiency, we sought to further clarify the regulatory role of CSTB on H3cs1 in the mouse brain. We found that CSTB functions as a chromatin-specific inhibitor of cathepsin L, suggesting that this protease is responsible for the generation of H3cs1 in the brain. CSTB modulates the levels of H3cs1 throughout postnatal brain development and mice lacking a functional *Cstb* gene exhibit elevated levels of H3cs1 until adulthood. Finally, even if the *Cstb*^–/–^ cerebellum shows altered expression of senescence-associated markers, further studies are warranted to confirm the presence of senescence in *Cstb*^–/–^ brain.

## Materials and methods

### Mouse model

The CSTB-deficient (*Cstb*^–/–^) mouse strain used in this study is 129S2/SvHsd5-*Cstb*^*tm*1*Rm*^, derived from the Jackson Laboratory strain 129-*Cstb*^*tm*1*Rm*^/J (stock no. 003486)^1^ (Pennacchio et al., 1998). Wild-type (*wt*) offspring from heterozygous matings were used as controls. Mice were genotyped for the *Cstb*^*tm*1*Rm*^ mutation using genomic DNA isolated from ear clippings and confirmed with DNA isolated from tail clippings following euthanasia. Mouse ages correspond to the following human developmental stages: P7 = early infancy, P14 = late infancy, P21 = childhood, P30 = adolescence, P120 = adulthood, based on age-dependent behavioral and hormonal signatures (Bell, 2018). The research protocols were approved by the Animal Ethics Committee of the State Provincial Office of Southern Finland (decisions ESAVI/10765/04.10.07/2015 and ESAVI/471/2019).

### Immunohistochemistry

P14 and older mice were intracardially perfused with heparin (0.16 mg/ml in PBS) and 4% paraformaldehyde (PFA) under pentobarbital-induced terminal anesthesia (100–200 mg/kg; Mebunat Vet, Orion). P7 mice were sacrificed without perfusion. Brains were bisected along the midline and immersion-fixed in 4% PFA for 48 h before cryoprotection in 30% sucrose, 0.05% sodium azide in TBS. Frozen coronal and sagittal sections of 40 μm were cut through the cerebrum and cerebellum, respectively, and collected as series of free-floating sections in 30% ethylene glycol, 15% sucrose, 0.05% sodium azide in TBS. For immunohistochemistry, tissue sections were (1) incubated with 50 mM ammonium chloride in TBS for 30 min, (2) blocked with 10% normal donkey serum, 1% BSA, 0.1–0.3% TritonX-100 in TBS for 1 h, (3) incubated with primary and secondary antibodies in 10% normal donkey serum, 0.3% TritonX-100 in TBS at 6°C, overnight or RT for 2 h, respectively, (4) counter-stained with DAPI (Abcam) for 5 min, and (5) mounted on chrome alum-gelatin coated microscopy slides using Fluoromount aqueous medium (Merck).

### Confocal microscopy and image analysis

Fluorescent tissue samples were imaged on a Zeiss LSM780 confocal system equipped with an Axio Observer.Z1 inverted microscope platform using ZEN 2.3 SP1 black acquisition software (Zeiss). The objectives utilized were a LD LCI Plan-Apochromat 25×/0.8 lmm korr DIC M27 (oil immersion), a Zeiss C Plan-Apochromat 40×/1.4 Oil DIC M27 and a Zeiss C Plan-Apochromat 63×/1.4 Oil DIC M27. DAPI, Alexa488 and Alexa594 were imaged with a 405 nm DPSS-laser, a 488 nm Argon laser and a 561 nm DPSS-laser and emission windows of 419–496 nm, 499–588 nm, and 588–733 nm, respectively. The pinhole was set to Airy 1 with unidirectional imaging and a pixel dwell time of 2 to 2.5 μs. No averaging was performed. Image analyses were carried out manually in ZEN 2.3 lite blue software (Zeiss) or Fiji/ImageJ 1.53 software except (1) H3cs1 and (2) Ki-67. Specifically, (1) the relative H3cs1 intensity per field was calculated by normalizing the fluorescence intensity of the H3cs1-positive cell nuclei [recognized automatically using the Fiji/ImageJ plugin for StarDist (Schmidt et al., 2018)] to the total nuclei count (counted manually using the DAPI field) and (2) Ki-67-expressing IBA1-positive cells were counted directly under the confocal microscope. For every biological replicate and condition, a minimum number of three random fields and 100 cells were analyzed. For standardization, images were obtained from the same areas in each cerebellar tissue sample.

### Protein extraction and western blotting

Mice were sacrificed by cervical dislocation. Following dissection, brains were bisected, rinsed with PBS and snap-frozen in liquid nitrogen. To prepare whole-tissue lysates, snap-frozen cerebella were lysed in 2% SDS, 12.5% glycerol, 60 mM Tris–HCl (pH 6.8) using a NG010 tissue grinder (Nippon Genetics), and further homogenized with a digital sonifier (Branson). Cytoplasmic and chromatin-bound protein extracts were prepared using a subcellular protein fractioning kit for tissues (Thermo Fisher Scientific, #87790). Specifically, (1) snap-frozen brains were homogenized with the loose pestle of a Dounce homogenizer (Jencons Scientific) for 13 strokes, filtered through a tissue strainer with a pore size of 250 μm and lysed in cytoplasmic extraction buffer as per manufacturer’s instructions. (2) Chromatin pellets were subsequently prepared from crude nuclei and lysed by micrococcal nuclease digestion for 30 min at room temperature. The presence of lysosomal proteins in the cytoplasmic extract was determined by immunoblotting for lysosomal marker LAMP1. Histone extracts were prepared using Histone Extraction Kit (Abcam, #ab113476). Protein concentrations were calculated using the BCA protein assay (Thermo Fisher Scientific). For western blotting, (1) protein samples were diluted in a sample buffer containing lithium dodecyl sulfate, glycerol, 50 mM DTT and SERVA Blue G250. (2) Protein samples were resolved using 4–12% Bis-Tris polyacrylamide gels in MES SDS running buffer (Bolt, Thermo Fisher Scientific). (3) Proteins were transferred to PVDF or nitrocellulose membranes with a pore size of 0.2 μm using TransBlot Turbo semi-dry system (Bio-Rad) in transfer buffer containing 39 mM glycine and 20% ethanol in 48 mM Tris (pH 9.2). (4) Target proteins were detected by enhanced chemiluminescence (ECL) using SuperSignal West Femto Maximum Sensitivity Substrate (Thermo Fisher Scientific) at antibody-dependent concentrations. (5) Molecular weights were calculated using two independent protein ladders. (6) For total protein normalization, membranes were stained with 0.1% amido black 10B (Thermo Fisher Scientific) and analyzed using Image Lab software (version 6.0, Bio-Rad).

### Enzymatic activity assays

Cathepsin L activity measurements were carried out using a substrate-based assay (Abcam, ab65306). Specifically, (1) assays were performed in the presence of 1.5 μM CA-074 (Merck) to prevent substrate cleavage by cathepsin B and (2) a standard curve with aminofluorocumarin (AFC, Abcam) was used to calculate AFC production from the fluorescent intensity readout of the assay. *In situ* β-galactosidase activity assays were carried out as previously described (Dimri et al., 1995). Specifically, (1) PFA-fixed free-floating cerebellar sections were incubated in staining solution, containing 5 mM potassium ferricyanide, 5 mM potassium ferrocyanide, 2 mM magnesium chloride, 150 mM sodium chloride and 1 mg/mL X-gal in 40 mM citric acid—sodium phosphate buffer (pH 6) for 3 h and 30 min at 37°C, and (2) bright-field microscopy images (four random fields per biological replicate and experimental condition) were captured using a Zeiss Axio Imager M2 microscope equipped with 20× objective and Axiocam 503 camera (Zeiss) and analyzed using Fiji/ImageJ 1.53 software.

### Primary antibodies

Rabbit α-H3cs1 [Cell Signaling Technologies, western blot (WB) 1:1,000, immunohistochemistry (IHC) 1:500, RRID:AB_2797961], mouse α-NeuN (Thermo Fisher Scientific, IHC 1:500, RRID:AB_2298772), rat α-GFAP (Thermo Fisher Scientific, IHC 1:300, RRID:AB_2532994), goat α-Olig2 (R and D Systems, IHC 1:1,000, RRID:AB_2157554), goat α-IBA1 (Novus, IHC 1:500, RRID:AB_521594), rabbit α-histone H3 (Abcam, WB 1:15,000, RRID:AB_302613), mouse α-GAPDH (Abcam, WB 1:10,000, RRID:AB_2107448), rat α-LAMP1 (DSHB, WB 1:1,000, RRID:AB_2134500), goat α-cathepsin B (R and D Systems, WB 1:2,000, RRID:AB_2086949), goat α-cathepsin F (Thermo Fisher Scientific, WB 1:2,000, RRID:AB_2576799), mouse α-cathepsin L (Abcam, WB 1:1,000, RRID:AB_305417), rabbit α-lamin B1 (Abcam, WB 1:2,000, RRID:AB_443298), rabbit α-p21^cip1^ (Abcam, IHC 1:500, RRID:AB_2734729), rabbit α-γH2AX (Abcam, WB 1:3,000, IHC 1:10,000, RRID:AB_1640564), rabbit α-IBA1 (Wako, IHC 1:4,000, RRID:AB_839504), rat α-Ki-67 (Thermo Fisher Scientific, IHC 1:500, RRID:AB_10853185).

### Real-time PCR

Real-time-qPCR assays were performed in accordance with MIQE guidelines (Bustin et al., 2009). Total RNA was isolated from cerebellum with NucleoSpin RNA Plus kit (Macherey-Nagel) and reverse-transcribed with iScript cDNA synthesis kit (BioRad). RT-qPCR were performed on a CFX96 Real-Time System (Bio-Rad) using TaqMan Fast Advanced Master Mix (Applied Biosystems). The following TaqMan probes were used for target amplification: *Cstb* (Mm00432769_m1), *Lmnb1* (Mm00521949_m1), *Ywhaz* (Mm03950126_s1), and *Rpl13* (Mm02526700_g1). *Cstb* and *Lmnb1* expression data was normalized to that of both *Ywhaz* and *Rpl13*, chosen due to their stable expression in contexts of neural development (Daura et al., 2021) and neurodegeneration (Rydbirk et al., 2016), respectively. Relative gene expression was calculated using the 2^–ΔCt^ method.

### Statistics

The experimental unit (n) in the analyses was one individual animal, except in the nuclear area measurements, where the experimental unit was defined as one individual cell. Statistical analyses were carried out with GraphPad Prism v8.4.2.679. Comparisons between two experimental conditions were made using Student’s *t*-test with Welch’s correction. Comparisons between three or more experimental conditions were made using one-way ANOVA with Sidak correction or two-way ANOVA with Tukey’s correction. The normality assumption of ANOVA was assessed with the Shapiro–Wilk test and by manually inspecting the distribution of ANOVA residuals. In datasets not conforming to the normal distribution, comparisons between three or more experimental conditions were made using Kruskal–Wallis test with Dunn’s correction.

Differences between groups were considered statistically significant when *P* < 0.05.

## Results

### Postnatal brain development and maturation involve chromatin targeting of cathepsin L and histone H3 tail proteolysis

We previously showed that murine neural precursors cultured *in vitro* undergo limited proteolysis of the N-terminal tail of H3cs1 upon induction of differentiation and that CSTB modulates the cleavage event, with altered cleavage in CSTB-deficient cells (Daura et al., 2021). Moreover, we reported that H3cs1 during *in vitro* neurogenesis is mediated by cysteine protease cathepsins B and L. The increase in H3cs1 was also evident in embryonic mouse brain extracts. Here we investigated the significance of this mechanism in postnatal mouse brain.

**FIGURE 1:**
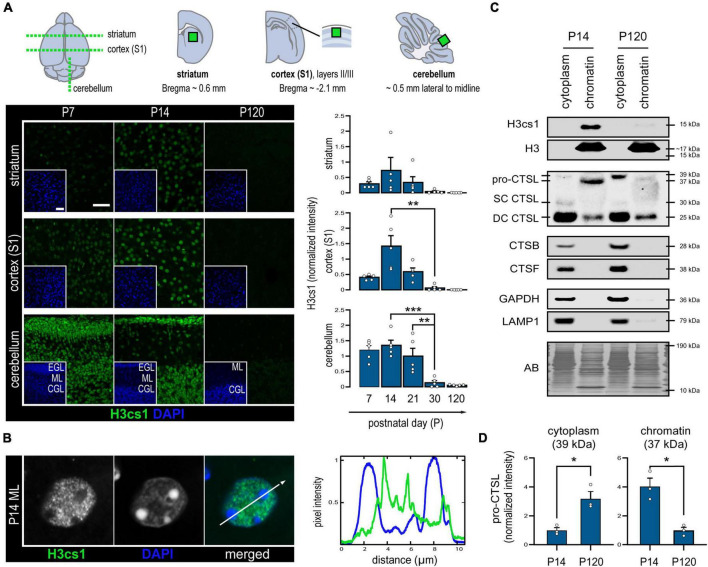
Levels of H3cs1 and chromatin-bound cathepsin L in the wild-type mouse brain. **(A)** Representative confocal microscopy images and quantitative analysis of H3cs1 immunohistochemical staining from P7 to P120 in the indicated brain regions (*n* = 5 mice/timepoint). The charts depict mean H3cs1 intensity ± SEM. A schematic representation of the sampling strategy is provided. Cortex (S1) = primary somatosensory cortex, EGL = external granular cell layer, ML = molecular layer, CGL = cerebellar granular cell layer. Scale bar = 40 μm. **(B)** Line profile of fluorescence intensity from a representative cell nucleus in the P14 brain, highlighting opposing staining patterns of H3cs1 and DAPI. **(C)** Western blot detection of H3cs1, total histone H3 and cysteine proteases cathepsin L (CTSL), cathepsin B (CTSB) and cathepsin F (CTSF) in lysosome-enriched cytoplasmic and chromatin-bound protein fractions from P14 and P120 mouse brains (*n* = 3 mice/timepoint). Four different CSTL forms were detected: cytoplasmic pro-CTSL (39 kDa), chromatin-bound pro-CTSL (37 kDa), single-chain mature CTSL (SC CTSL, 30 kDa) and double-chain mature CTSL (DC CTSL, 25 kDa). GAPDH and LAMP1 were used as cytoplasmic and lysosomal markers, respectively. AB = amido black. **(D)** Age-dependent changes in cytoplasmic pro-CTSL and chromatin-bound pro-CTSL content. Normalized intensity values are plotted as means ± SEM (*n* = 3 mice/timepoint). *P < 0.05, **P < 0.01, and ***P < 0.001.

We first asked whether histone tail cleavage occurs in a spatiotemporally regulated manner in the central nervous system. To characterize the distribution of H3cs1 in the wild-type mouse brain, we performed immunohistochemistry of serial brain sections at five timepoints spanning from early infancy (P7) to adulthood (P120). We quantified the intensity of H3cs1 in three anatomically distinct brain regions: the primary somatosensory cortex (S1), the striatum, and the cerebellum (Figure 1A). H3cs1 occurred at varying levels from P7 to P21, peaking at P14 in the primary somatosensory cortex and, to a lesser extent, in the striatum. In the cerebellum, we detected steadily high levels of H3cs1 from P7 to P21. On the contrary, H3cs1 was virtually absent at P30 and P120 in all brain regions. These observations indicate that H3cs1 normally occurs during postnatal mouse brain development and maturation.

In P14 brain, practically all NeuN positive neurons and GFAP positive astrocytes contained H3cs1 in their nuclei (Supplementary Figure 1). Instead, Olig2 positive oligodendrocyte and the IBA1 positive microglia/macrophage lineages showed both positive and negative cells. These data imply that H3cs1 is subjected to cell-type specific regulation *in vivo*. Examination of individual cell nuclei revealed that H3cs1 is largely excluded from DAPI-dense heterochromatin clusters (Figure 1B). This is in line with previous reports, which indicate that H3cs1 localizes in open chromatin regions implicating its regulatory role in gene expression (Santos-Rosa et al., 2009; Cheung et al., 2021).

We next sought to uncover the cysteine proteases, which cleave H3cs1 in mouse brain. We first analyzed subcellular cytoplasmic and chromatin fractions from P14 and P120 brains of *wt* mice by western blotting. In line with the immunohistochemistry analysis (Figures 1A,B), H3cs1 was clearly visible in the P14, but not in P120 chromatin fractions (Figure 1C). We did not detect the 15 kDa H3cs1 band in chromatin fractions using an antibody against the H3 C-terminus (Figure 1C), but a faint band corresponding to H3cs1 was observed in histone extracts from P14 brain (Supplementary Figure 2), indicating that H3cs1 only corresponds to the small proportion of the total H3cs1 in the *wt* mouse brain. We next probed the cytoplasmic and chromatin fractions with antibodies against three cysteine proteases with a previously reported nuclear localization, cathepsin L (Goulet et al., 2004), cathepsin B (Hämälistö et al., 2020), and cathepsin F (Maubach et al., 2008). Cathepsins B and F were only observed in lysosome-enriched cytoplasmic fractions, whereas cathepsin L was also present in chromatin fractions (Figure 1C). In cytoplasmic fractions, we detected three cathepsin L signals matching the pro-cathepsin L (39 kDa), and the mature single-chain (30 kDa) and double-chain (25 kDa) forms of cathepsin L (Ishidoh et al., 1998). In chromatin fractions, we detected the mature double-chain cathepsin L and a shorter, 37 kDa form of pro-cathepsin L in accordance with previous studies showing that the nuclear targeting of cathepsin L is accomplished through the alternative translation of the *Ctsl* mRNA into a shorter pro-cathepsin L devoid of a lysosome import signal (Goulet et al., 2004; Burton et al., 2017). The chromatin-associated shorter pro-cathepsin L was more abundant at P14 than at P120, whereas the cytoplasmic, longer pro-cathepsin L was more abundant at P120 than at P14 (Figures 1C,D). These findings imply that the subcellular targeting of *de novo* synthesized cathepsin L shifts from nuclear to cytoplasmic (lysosomal) in the adult brain, suggesting a role for nuclear cathepsin L in brain development and maturation. No evident differences between P14 and P120 were detected in the amount of either cytoplasmic or chromatin-associated mature double-chain forms of cathepsin L.

### Cystatin B-deficiency leads to increased chromatin-associated cathepsin L activity in brain

**FIGURE 2:**
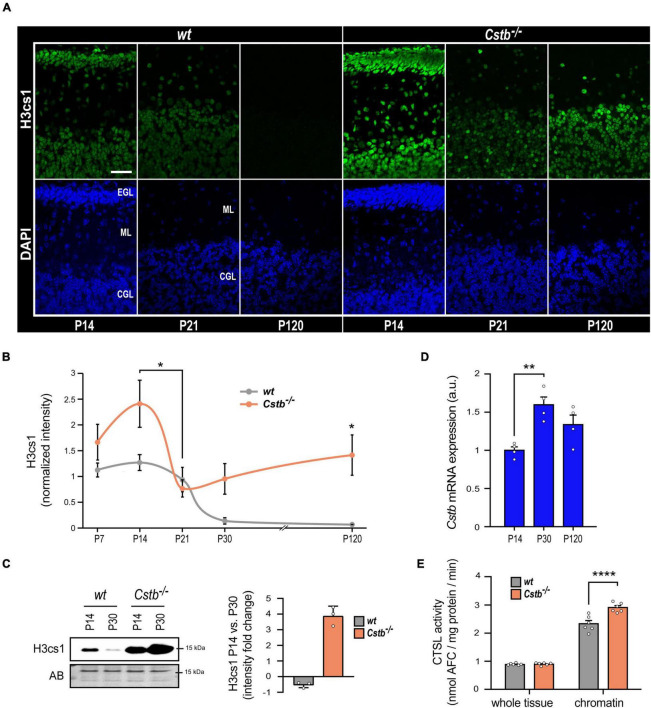
The effect of CSTB-deficiency on H3cs1 levels and cathepsin L activity. **(A)** Representative confocal microscopy images of H3cs1 immunohistochemical staining in the cerebellum of P14, P21, and P120 *wt* and *Cstb*^–/–^ mice. EGL = external granular cell layer, ML = molecular layer, CGL = cerebellar granular cell layer. Scale bar = 40 μm. **(B)** Quantitative analysis of H3cs1 immunohistochemical staining in the cerebellum of P7, P14, P21, P30, and P120 *wt* and *Cstb*^–/–^ mice (*n* = 5 mice/timepoint). The charts depict H3cs1 content as mean ± SEM. The pattern of H3cs1 intensity in the examined timepoints is significantly increased in *Cstb*^–/–^ samples (*P* < 0.0001). **(C)** Western blot detection of H3cs1 in cerebellar lysates of P14 and P30 *wt* and *Cstb*^–/–^ mice. The bar chart depicts fold changes in H3cs1 levels from P14 to P30 in *wt* and *Cstb*^–/–^ mice. Normalized intensity values are plotted as means ± SEM (*n* = 3 mice/timepoint and genotype). AB = amido black. **(D)** RT-qPCR analysis of *Cstb* mRNA expression in postnatal brain of *wt* mice plotted as means ± SEM (*n* = 4 mice/timepoint). **(E)** Bar charts depicting the enzymatic activity of cathepsin L in whole brain lysates and in chromatin-bound protein fractions of *wt* and *Cstb*^–/–^ P30 mice. Aminofluorocumarin production values (nmol AFC/min) are normalized to the total protein input and plotted as means ± SEM (*n* = 6 mice/timepoint and genotype). *P < 0.05, **P < 0.01, and ****P < 0.0001.

We previously showed that overexpression of CSTB abolished H3cs1 in differentiating mouse neuronal precursors derived from *Cstb*^–/–^ mice indicating that CSTB acts as a modulator of H3cs1 tail proteolysis in neural cells (Daura et al., 2021). Here, we investigated the impact of CSTB deficiency on H3cs1 tail proteolysis in the postnatal mouse brain. We concentrated the analysis to the *Cstb*^–/–^ mouse cerebellum for its early onset and striking neuronal degeneration and glial activation (Pennacchio et al., 1998; Tegelberg et al., 2012). We performed immunohistochemical staining of H3cs1 in serial cerebellar sections from *Cstb*^–/–^ mice at five postnatal timepoints (P7, P14, P21, P30, and P120) covering postnatal cerebellar development and maturation and compared them to the *wt*. The pattern of H3cs1 intensity in the examined timepoints varied significantly between the genotypes (Figures 2A,B). In *wt* cerebellum, the H3cs1 level decreased from P14 to P120, whereas in *Cstb*^–/–^ cerebellum, H3cs1 initially decreased from P14 to P21, but then increased from P21 onward (Figures 1A, 2A,B). A western blot analysis of cerebellar lysates showed that, in *wt* mice, the level of H3cs1 decreased by half, whereas in *Cstb*^–/–^ mice, it increased fourfold from P14 to P30 (Figure 2C). As an increase in *Cstb* mRNA expression is temporally correlated with a decrease in H3cs1 during *in vitro* differentiation (Daura et al., 2021), we asked the question whether the same correlation is also seen in postnatal brain and analyzed *Cstb* mRNA expression in P14, P30, and P120 *wt* mouse cerebellum by qPCR. We detected an increase in the levels of *Cstb* mRNA from P14 to P30 (Figure 2D), concomitant with the decrease in H3cs1 levels (Figures 2A–C) implying that CSTB functions as negative regulator of H3cs1 also *in vivo*.

Next, we sought to clarify whether the regulatory function of CSTB on H3cs1 could be mediated through its inhibitory effect on cathepsin L activity. We measured the enzymatic activity of cathepsin L in whole tissue lysates and in chromatin-associated protein fractions from *wt* and *Cstb*^–/–^ mice brains at P30 (Figure 2E). At the whole tissue level, cathepsin L activity did not differ between the genotypes, but it was higher in chromatin fraction of *Cstb*^–/–^ than of *wt* brain (Figure 2E). These findings suggest that CSTB serves as a chromatin-specific inhibitor of cathepsin L *in vivo.*

### Altered presentation of senescence-associated markers in cystatin B-deficient cerebellum

Histone H3 tail cleavage by cathepsin L intensifies during cellular senescence. Importantly, the ectopic overexpression of cleaved histone H3 is sufficient to induce senescence in fibroblasts and melanocytes (Duarte et al., 2014). We investigated whether sustained H3cs1 is associated with cellular senescence in the *Cstb*^–/–^ mouse brain. We focused the analysis on the cerebellum, the brain region that presents the highest overall H3cs1 both in *wt* and in *Cstb*^–/–^ mice (Figures 1A, 2A).

**FIGURE 3:**
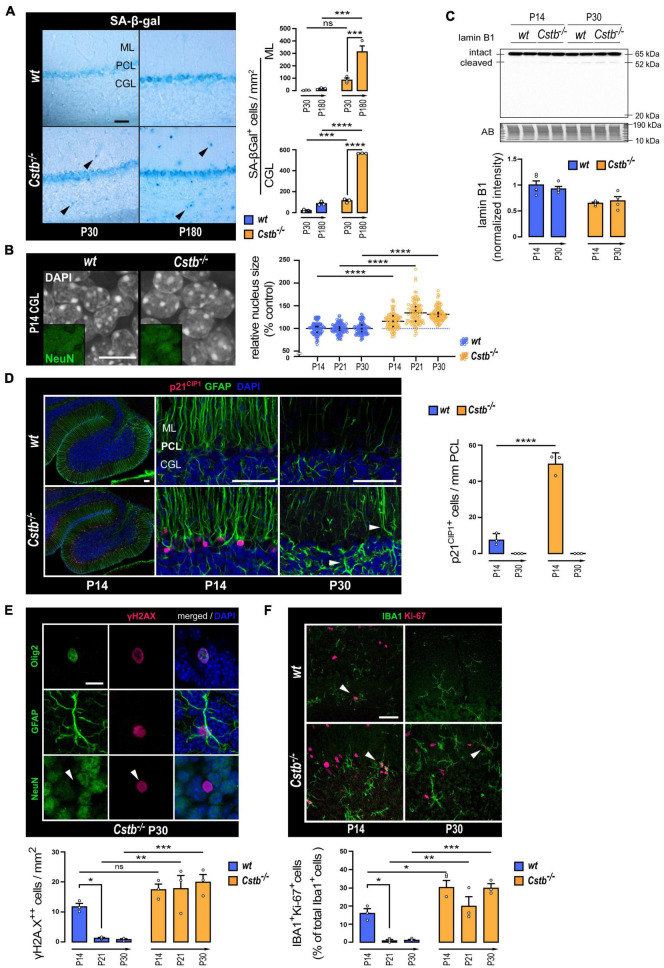
Markers linked to cellular senescence in the cerebellum of pre-symptomatic CSTB-deficient mice. **(A)** Representative bright-field microscopy images and quantitative analysis of SA-β-gal in the cerebellum of early symptomatic (P30) and late-symptomatic (P180) *Cstb*^–/–^ mice with age-matched *wt* littermates as controls. The charts depict SA-β-gal-positive cell densities as mean ± SEM (*n* = 3 mice/timepoint and genotype). Scale bar = 40 μm. **(B)** Representative confocal microscopy images, and nuclear areas of granule neurons (NeuN-positive cells of the CGL) in the developing cerebellum of *wt* and *Cstb*^–/–^ mice. The chart depicts nuclear area in relation to age-matched *wt* controls as mean ± SEM (*n* = 108 nuclei/timepoint and genotype). Average values for each animal are indicated with black dots. Scale bar = 10 μm. **(C)** Western blot detection of lamin B1 in cerebellar lysates of *wt* and *Cstb*^–/–^ mice at P14 and P30. The bar chart illustrates intact lamin B1 protein levels. Normalized intensity values are plotted as means ± SEM (*n* = 4–5 mice/timepoint and genotype). **(D)** Representative confocal microscopy images and quantitative analysis of p21^CIP1^ immunoreactivity in the Bergmann glia (GFAP-positive cells of the Purkinje cell layer) of the developing cerebellum of *wt* and *Cstb*^–/–^ mice. The chart depicts linear densities of p21^CIP1^-positive cells as mean ± SEM (*n* = 3 mice/timepoint and genotype). Hypertrophic GFAP-positive processes denoting astrogliosis in the P30 *Cstb*^–/–^ cerebellum are shown with white arrowheads. Scale bars = 50 μm. **(E)** Representative confocal microscopy images of P30 *Cstb*^–/–^ cerebellar sections stained for γH2AX, GFAP, Olig2, and NeuN and quantitative analysis of pan-nuclear γH2AX-positive cells in the developing cerebellum of *wt* and *Cstb*^–/–^ mice. The chart depicts pan-nuclear γH2AX-positive cell densities as mean ± SEM (*n* = 3 mice/timepoint and genotype). Scale bar = 10 μm. **(F)** Representative confocal microscopy images and quantitative analysis of IBA1/Ki-67 staining of the developing cerebellum of *wt* and *Cstb*^–/–^ mice. The chart depicts the proportion of proliferative microglia (Ki-67/IBA1-double positive cells/IBA1-positive cells) as means ± SEM (*n* = 3 mice/timepoint and genotype). Scale bar = 40 μm. Abbreviations: SA-β-gal, senescence-associated lysosomal β-galactosidase activity; ML, molecular layer; PCL, Purkinje cell layer; CGL, cerebellar granular cell layer; AB, amido black; ns, not significant. *P < 0.05, **P < 0.01, ***P < 0.001, and ****P < 0.0001.

We first evaluated the acidic lysosomal beta-galactosidase (SA-β-Gal) activity, which has been considered an archetypical senescence-associated biomarker (Dimri et al., 1995), to elucidate whether mice presented signs of cellular senescence at the emergence of neurodegeneration (P30) or in the presence of significant neuron loss (P180). When compared to *wt* mice, an increase in the number of SA-β-Gal positive cells was detected in *Cstb*^–/–^ mice (Figure 3A). The increase was observed in the granule cell layer at P30 and at P180, and in the molecular layer at P180. Moreover, the SA-β-Gal positive cell count increased from early symptomatic P30 to late symptomatic P180 *Cstb*^–/–^ cerebellum.

To investigate whether CSTB-deficient mouse brains show signs of cellular senescence before the first signs of neuron loss and onset of myoclonus at P30, we focused our further analyses on the time window from the P14 to P30. H3cs1 is highest in both genotypes at P14, the timespoints when the first signs of gliosis appear in *Cstb*^–/–^ brain (Tegelberg et al., 2012), while at P30, H3cs1 is downregulated to very low levels of *wt* adult brain but remains high in *Cstb*^–/–^ brain (Figure 2A). To evaluate whether *Cstb*^–/–^ cerebellum displays enlarged cell nuclei, another well-established hallmark of cellular senescence (Mitsui and Schneider, 1976), we measured in tissue sections of *Cstb*^–/–^ and *wt* mice the nuclear area of cerebellar granule cells, the cell type that show marked progressive loss in *Cstb*^–/–^ brain (Pennacchio et al., 1998). We found that cerebellar granule cell nuclei were on average 26% larger in *Cstb*^–/–^ than in age-matched *wt* controls (Figure 3B). This phenotype was observed at P14, P21, and P30, indicative of changes in nuclear architecture manifesting at least 2 weeks prior to the symptomatic onset of the disease. However, lamin B1, a major component of the nuclear lamina, whose downregulation is usually linked to nuclear swelling during cellular senescence (Freund et al., 2012), showed no differences between the genotypes either in mRNA or protein levels, nor in the cleavage pattern (Figure 3C and Supplementary Figure 3).

Next, we performed a series of immunohistochemical analyses to look for expression changes in cyclin-dependent kinase (CDK) inhibitors, a group of proteins with an essential role in inducing the stable proliferative arrest characteristic of cellular senescence (reviewed in Martínez-Zamudio et al., 2017). CDK inhibitors p27 and p16^INK4a^ showed no differences between the genotypes (data not shown). Increased expression of p21^CIP1^ was observed in glial fibrillary acidic protein (GFAP) positive Bergmann glia at P14 (Figure 3D). In *wt* mice, p21^CIP1^ positive cells occurred in low frequency, suggesting that a subpopulation of these radial glia exits in cell cycle during normal cerebellar development. In *Cstb*^–/–^ mice, the number of p21^CIP1^ positive cells was increased sixfold in comparison to *wt* controls (Figure 3D). However, by P30, no p21^CIP^ positive cells we observed in either genotype.

To find out whether *Cstb*^–/–^ mice present altered or persistent DNA-damage responses, another characteristic feature of senescent cells (Sedelnikova et al., 2004; Collin et al., 2018), we analyzed the level and staining pattern of histone variant H2AX phosphorylated at Ser139 (γH2AX), a DNA double strand break response marker (Kuo and Yang, 2008). Western blot analysis of whole-tissue lysates showed that the levels of γH2AX decreased significantly in both genotypes after the second postnatal week, in accordance with previous findings (Barral et al., 2014), with no apparent differences between genotypes (Supplementary Figure 4A). The number of cells presenting a pan-nuclear γH2AX immunoreactivity in *wt* cerebellum decreased after P14 (Figure 3E and Supplementary Figure 4B). On the contrary, in *Cstb*^–/–^ mice, the pan-nuclear γH2AX-positive cell count was constantly elevated in all three timepoints analyzed (Figure 3E and Supplementary Figure 4B). Pan-nuclear γH2AX responses mediate DNA damage events coupled to cell death by apoptosis (Ding et al., 2016). Accordingly, we observed that, in many of these cells, the γH2AX signal organized into an apoptotic ring conformation, an early marker of apoptosis (Solier and Pommier, 2009; Supplementary Figure 4C). Further analysis revealed that the cells displaying pan-nuclear γH2AX immunoreactivity were either Olig2 positive oligodendrocytes or GFAP positive astrocytes, but not NeuN positive neurons (Figure 3E).

In *Cstb*^–/–^ mouse brain, microglia become activated before the onset of clinical symptoms (Tegelberg et al., 2012). Therefore, this cell type is likely to play an important, yet so far unknown role in the disease pathogenesis. A growing body of evidence indicates that many chronic neurodegenerative diseases involve reactivation of microglial proliferation (Olmos-Alonso et al., 2016), a pathologic feature that eventually elicits microglial senescence by proliferative exhaustion (Hu et al., 2021). To assess the potential effect of CSTB-deficiency on microglia senescence through proliferative exhaustion (Hu et al., 2021), we quantified the proportion of IBA1 positive microglia expressing the proliferation antigen Ki-67 in cerebellar tissue sections from *wt* and *Cstb*^–/–^ mice (Figure 3F). In *wt* cerebellum, the percentage of dividing microglia decreased from approximately 16% at P14 to 1% at P21 and at P30, in line with previous findings (Nikodemova et al., 2015). In *Cstb*^–/–^ cerebellum, the proliferation of microglia was approximately twofold higher than in age-matched *wt* controls at P14 and presented no significant decline at P21 or P30.

## Discussion

We have previously shown that CSTB modulates cysteine cathepsin-mediated H3cs1 tail cleavage in mouse neurosphere-derived cells (Daura et al., 2021). In this follow-up study, we examined the occurrence of this mechanism in the context of mouse postnatal brain development and maturation.

We show that H3cs1 is spatiotemporally regulated in postnatal brain. Three areas were chosen for closer inspection in the *wt* mouse brain: cerebellum and primary somatosensory cortex for their previously described early signs of pathological changes in *Cstb*^–/–^ mouse (Pennacchio et al., 1998; Tegelberg et al., 2012) and striatum as a large anterior, subcortical area. Peaking of H3cs1 at P14 in the cortex and striatum coincides with a period characterized by fast growth of neurons with maturation of somata and pruning of dendrites and axons (Romand et al., 2011). As H3cs1 has been previously implicated in transcriptional regulation (Santos-Rosa et al., 2009; Cheung et al., 2021), the observed pattern of H3cs1 may reflect the increase in expression of genes involved in fast growth. The high H3cs1 intensity in cerebellum without a clear peak from P7 to P21 is likely to be associated with the relatively late development of this brain region (Butts et al., 2014). In the *Cstb*^–/–^ mouse brain, where we focused the analysis on cerebellum, the pattern of H3cs1 expression was significantly different from that in *wt* mice, remaining high still at 4 months. These data from corroborate our previous *in vitro* data and suggest CSTB acts as negative regulator of H3cs1 also *in vivo*.

Our previous results, based on analysis of whole cell lysates of cultured cells, suggested that cysteine protease cathepsins B and L mediate histone cleavage during *in vitro* neurogenesis (Daura et al., 2021). Here we focused the analysis on cytoplasmic and chromatin-bound protein extracts from mouse brain tissue. In *wt* brain, of the three cathepsins with reported nuclear localization only cathepsin L was found in the chromatin fraction. We further showed that the subcellular localization of pro-cathepsin L is age-dependent in mouse brain. Chromatin-bound pro-cathepsin L is present at high levels during the period of developmental maturation at P14, but at low levels in adult brain, this pattern correlating with the intensity of H3cs1. These findings are in line with cathepsin L being the most extensively documented protease with the ability to cleave histone tails both *in vitro* and *in vivo* (Duncan et al., 2008; Adams-Cioaba et al., 2011) and strongly suggest that cathepsin L is responsible for the generation of H3cs1 in the postnatal mouse brain.

Our finding in cerebella of *Cstb*^–/–^ mice, where, compared to *wt*, increased H3cs1 intensity correlates with increased chromatin-associated cathepsin L activity at P30, supports the previously reported role of CSTB as a regulator of H3cs1 through cathepsins in neural cells (Daura et al., 2021). Interestingly, we did not observe genotype-specific alterations in cathepsin L activity at the whole tissue level, suggesting that CSTB specifically modulates the chromatin-bound pool of cathepsin L in the mouse brain. These findings are in contrast with studies in human lymphoblastoid cells (Rinne et al., 2002) and in murine bone marrow-derived macrophages (Maher et al., 2014), where CSTB-deficiency led to a more pronounced increase in cathepsin L activity that was readily detected in whole cell lysates. Importantly, a complete loss of nuclear cathepsin L activity has been associated with a global rearrangement of epigenetic marks (Bulynko et al., 2006), highlighting the profound implications of this protease in chromatin function. Conversely, knocking out *Cstb* in macrophages did not result in large-scale chromatin alterations (Maher et al., 2014), suggesting that CSTB is not an essential nuclear cathepsin L inhibitor in this cell type. In the light of these data, our findings raise the intriguing possibility that CSTB serves as a critical modulator of chromatin-associated cathepsin L specifically in the neural lineage, an interpretation that is further supported by the predominantly neurological effects of CSTB-deficiency in mice and in humans (Pennacchio et al., 1998; Mancini et al., 2016; Lehesjoki and Kälviäinen, 2020).

During embryonic stem cell differentiation, canonical histones H3.1 and H3.2 are preferably cleaved by cathepsin L over the replication-independent histone variant H3.3 (Duncan et al., 2008). On the other hand, the ectopic expression of H3.3cs1, but not that of H3.1cs1, triggered cellular senescence in fibroblasts and melanocytes (Duarte et al., 2014). In the mouse brain, replication-independent histone dynamics gain a crucial role after approximately second postnatal week, enabling life-long chromatin plasticity in post-mitotic cell populations (Maze et al., 2015). Due to the low abundance of H3cs1 in brain tissue we were not able to identify whether there is any preference in the histone isoforms cleaved by cathepsin L in postnatal brain. It is though tempting to speculate that the CSTB-mediated repression of H3cs1 after the second postnatal week prevents its chromatin integration by replication-independent mechanisms and thus also prevents triggering cellular senescence in brain (Duarte et al., 2014). We explored this hypothesis by investigating whether the sustained H3cs1 in *Cstb*^–/–^ cerebellum is associated with cellular senescence through analysis of several senescence-associated markers.

We detected accumulation of SA-β-Gal-positive cells in cerebella of *Cstb*^–/–^ mice, evident in the granule cell layer already at P30 and significantly increased in 6-month-old animals. We also detected a sixfold increased expression of CDK inhibitor p21^CIP1^, another marker linked to cellular senescence, in Bergmann glia of P14 *Cstb*^–/–^ cerebellum compared to *wt*. It should be noted, however, that during neuronal development some postmitotic neurons, notably Purkinje cells, express both SA-β-Gal and p21^CIP1^ without subsequent senescence or apoptosis, and the reliability of these senescence markers in the context of postmitotic neurons have been questioned (de Mera-Rodriguez et al., 2019, 2021). Indeed, the Purkinje cells in our experiments were positive for SA-β-Gal in both genotypes. Senescent cells usually display significant morphological changes, including nuclear swelling accompanied with downregulation of lamin B1, a major component of nuclear lamina (Freund et al., 2012). *Cstb*^–/–^ cerebellar granule cells present with enlarged cell nuclei, but we could not detect any changes in lamin B1 expression or cleavage pattern.

We also studied the expression of DNA double-strand break marker, phosphorylated H2AX (γH2AX), and found astroglial cells and oligodendrocytes positive for γH2AX in P14 cerebellum. Concordant with this, H2AX phosphorylation has been reported to occur during cerebellar development, peaking at P5-P10, and declining after P15 (Barral et al., 2014). While in *wt*, the number of γH2AX positive cells decline as expected after P14, in *Cstb*^–/–^ cerebellum, the number stays high. Given that γH2AX is a pleiotropic molecule with an array of yet not fully understood functions in the brain, including response to oxidative damage and apoptotic signaling (Merighi et al., 2021), the significance of its continued expression beyond P14 in *Cstb*^–/–^ cerebellum should be explored in further studies. Interestingly, apoptotic cell death is one of the major pathological signs in *Cstb*^–/–^ cerebellum (Pennacchio et al., 1998) where oxidative damage has even been described, although not on DNA level (Lehtinen et al., 2009). Of note, oxidative stress is also a common inducer of senescence (Sahu et al., 2022).

Microglia proliferate until P14 after which they experience extensive changes in their gene expression, morphology, and functions, which calls for major changes in epigenetic profile (Nikodemova et al., 2015). Aberrant continued proliferation is shown to lead to senescence through proliferative exhaustion (Hu et al., 2021). Our data indicate that in *Cstb*^–/–^ cerebellum, contrary to *wt*, microglial proliferation is not downregulated after P14, which could indicate failure to attain the adult epigenetic signatures. A recent study showed that transient accumulation of H3cs1 during macrophage differentiation keeps chromatin primed for further remodeling after H3cs1 is removed (Cheung et al., 2021). Here, we show that in mouse brain, H3cs1 accumulates during postnatal period, when transcriptional programs involved in constructing and shaping the brain are replaced by those involved in operating the nervous system, like neuronal activity and plasticity (Liscovitch and Chechik, 2013). This suggests that H3cs1 is important for the regulation of the genes that govern the latter stages of brain development, and inadequate repression of H3cs1 tail cleavage by CSTB could lead to defective transition to epigenetic signatures of mature brain. However, CSTB-deficient microglia show activated phenotype already at P14 and further studies are needed to clarify the relation of these observations.

Combined, our previous and current findings indicate that CSTB prevents premature histone cleavage before neural stem cell differentiation (Daura et al., 2021), modulates the levels of H3cs1 throughout brain development and is an essential factor in suppressing H3cs1 after the second postnatal week until adulthood. This developmentally regulated mechanism could explain why the complete loss of CSTB disrupts embryonic brain development in humans, whereas residual cellular CSTB expression results in the milder phenotype of EPM1, with apparently normal early development and symptomatic onset in late childhood/early adolescence (Koskenkorva et al., 2011; Mancini et al., 2016; Lehesjoki and Kälviäinen, 2020). The question whether CSTB-deficiency triggers cellular senescence remains open. We looked for presence of several reported markers of cellular senescence in the maturing *Cstb*^–/–^ cerebellum, where we see increased levels of H3cs1. None of these showed the similar widespread distribution as H3cs1, except for the increase in nuclear size of cerebellar granule neurons, yet this occurred without accompanying lamin B expression, that is often present in senescent cells (Freund et al., 2012). Observing cellular senescence reliably in tissues, especially in context of post-mitotic cells, is challenging due to non-specificity and low sensitivity of commonly used markers (Gonzalez-Gualda et al., 2021). Further studies are thus needed to unravel the putative role of H3cs1-induced senescence associated with deficient CSTB function.

Taken together, our findings add to an increasing body of evidence indicating that cathepsin L and its nuclear targets play central roles in brain disease (Fei et al., 2018; Harpaz et al., 2022; Islam et al., 2022). Pharmacological targeting of these processes could have important therapeutic applications, and therefore should be the focus of future studies. Our data strongly suggest that CSTB functions as a chromatin-specific inhibitor of nuclear cathepsin L, and therefore clarify the neurophysiological function of CSTB.

## Data availability statement

The raw data supporting the conclusions of this article will be made available by the authors, without undue reservation.

## Ethics statement

This animal study was reviewed and approved by the Animal Ethics Committee of the State Provincial Office of Southern Finland (decisions ESAVI/10765/04.10.07/2015 and ESAVI/471/2019).

## Author contributions

ED: conceptualization, methodology, formal analysis, investigation, data curation, funding acquisition, writing—original draft, writing—review and editing, and visualization. ST: methodology, investigation, and writing—review and editing. PH: investigation. A-EL and TJ: project administration, writing—review and editing, and funding acquisition. All authors contributed to the article and approved the submitted version.

## Footnotes

1
https://www.jax.org/strain/003486
