# Supplementary material for: Cystatin B deficiency results in sustained histone H3 tail cleavage in postnatal mouse brain mediated by increased chromatin-associated cathepsin L activity
Source: Front Mol Neurosci. 2022 Nov 30;15:1069122. doi: 10.3389/fnmol.2022.1069122 (PMC9749902; doi:10.3389/fnmol.2022.1069122)
Supplement: Supplementary file 1 [file Data_Sheet_1.docx]

Cystatin B deficiency results in sustained histone H3 tail cleavage in postnatal mouse brain mediated by increased chromatin-associated cathepsin L activity - Supplementary Material


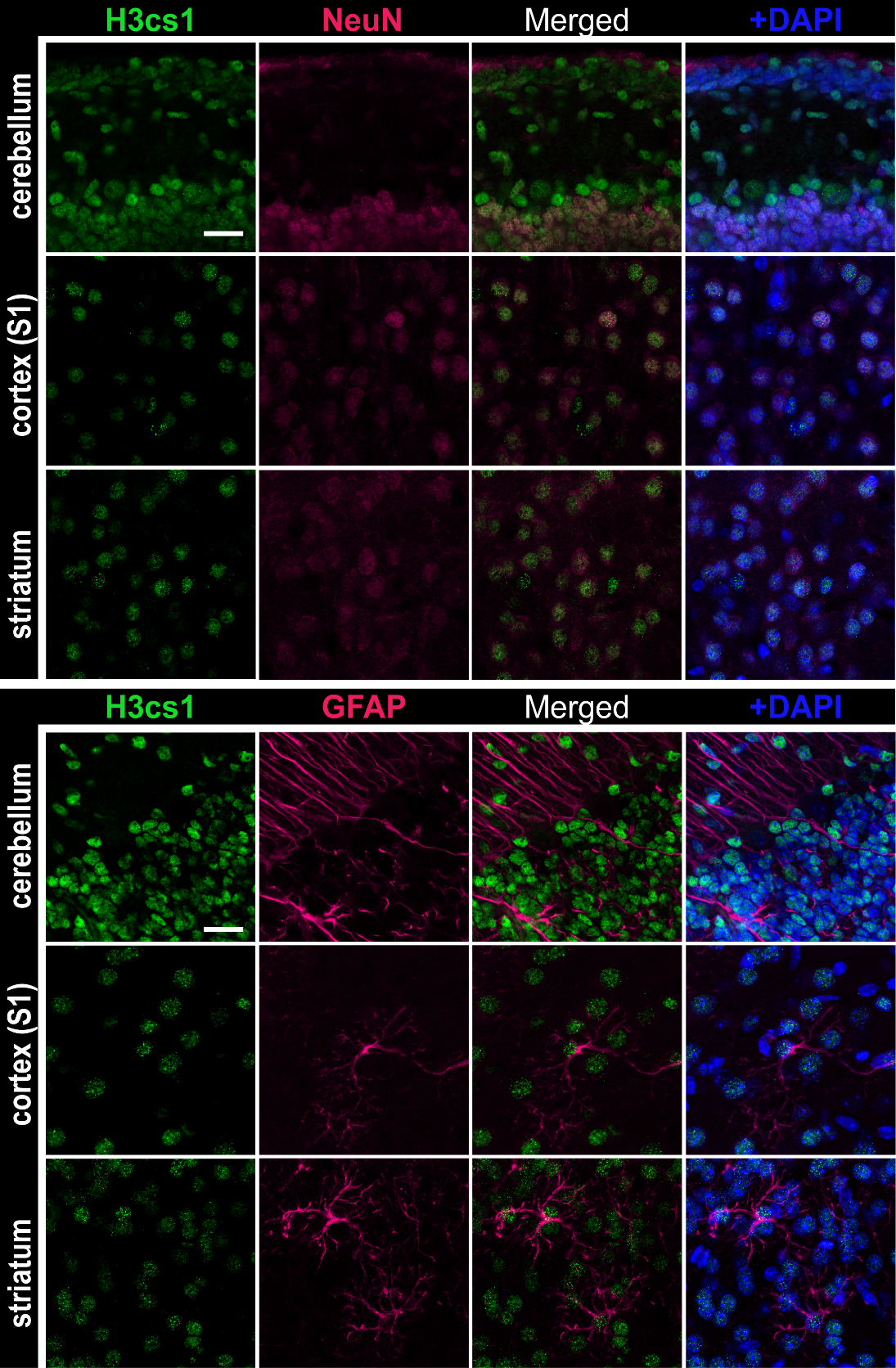


**
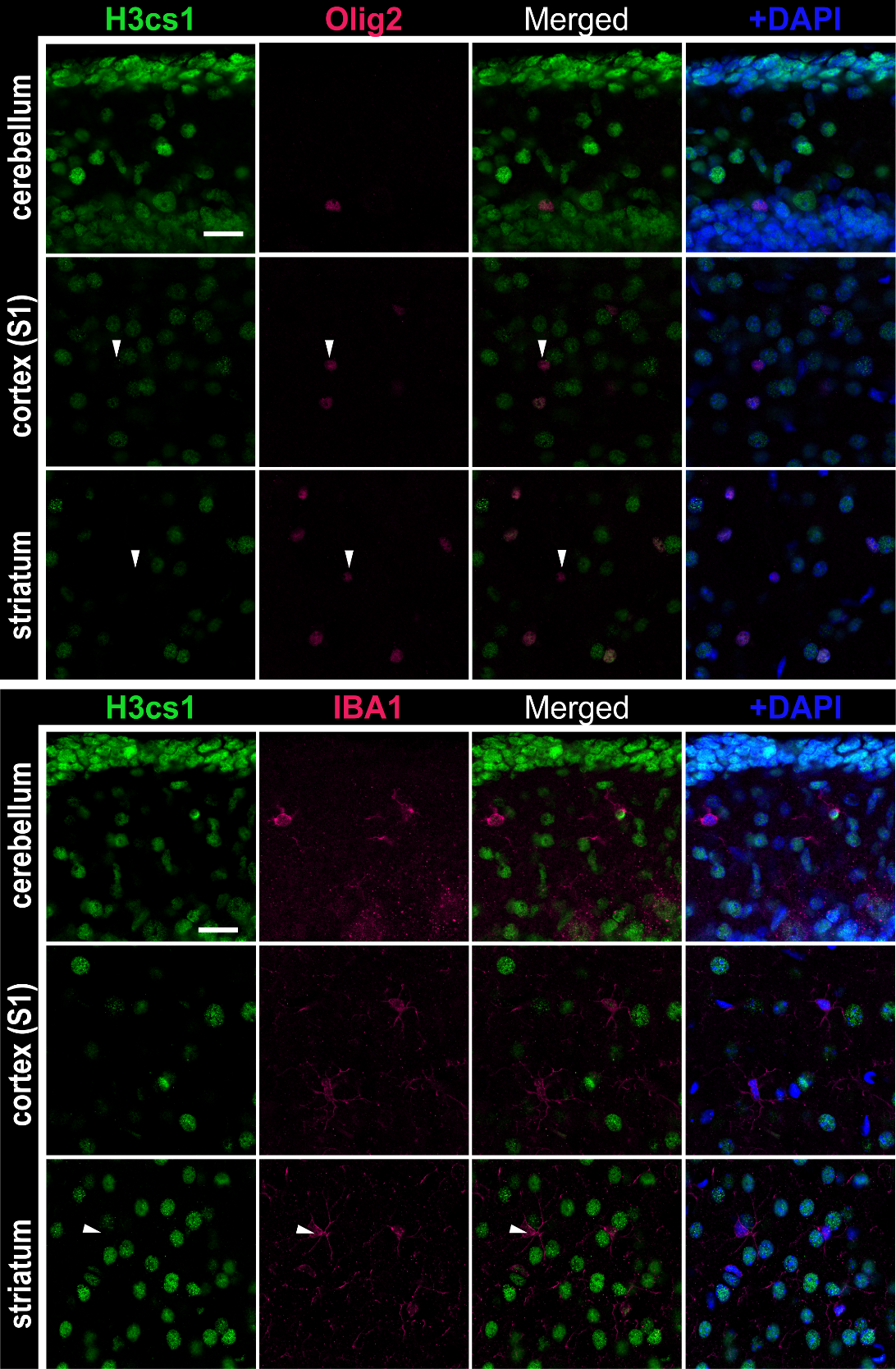
**

**Supplementary Figure 1.** Representative confocal microscopy images of P14 brain sections stained for H3cs1 and NeuN, GFAP, IBA1 or Olig2 in the cerebellum, the primary somatosensory cortex (S1) and the striatum. H3cs1 localizes in the nucleus of neurons, astrocytes, microglia and oligodendrocytes. H3cs1-negative cells (marked with white arrowheads) were only detected among microglia and oligodendrocytes.

**
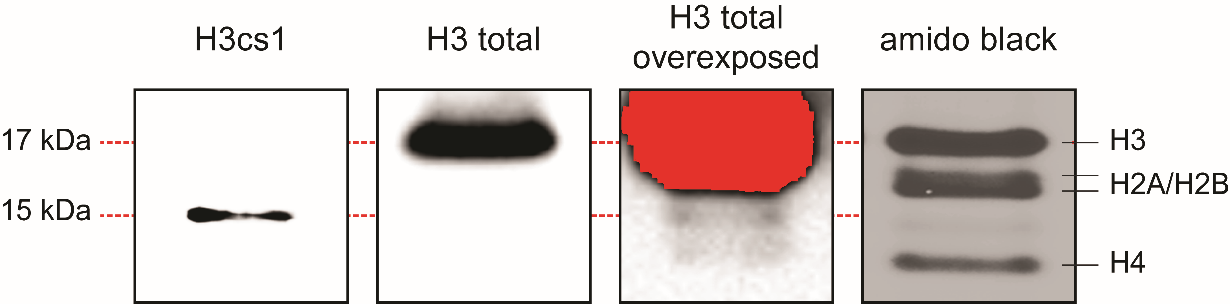
**

**Supplementary Figure 2.** H3cs1 is a low-abundance histone modification in the brain. Sequential western blot detection of H3cs1 and histone H3 in total histone extracts from the P14 mouse brain shows that H3cs1 represents a very small fraction of the histone H3 pool. Saturated pixels are shown in red.


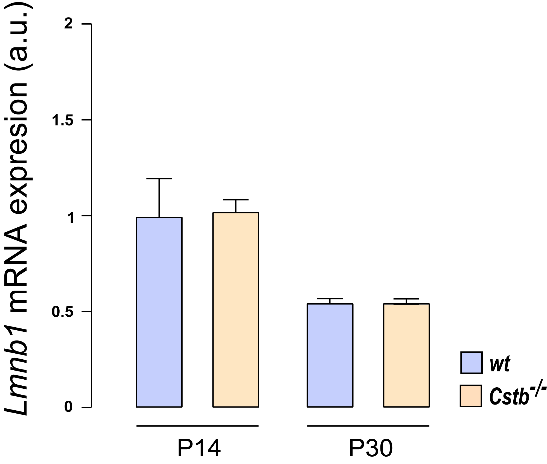


**Supplementary Figure 3.** The mRNA expression of lamin B1 in the postnatal cerebellum is not affected by CSTB deficiency. RT-qPCR analysis of Lmnb1 mRNA expression in cerebella of wt and Cstb-/- mice at P14 and P30 plotted as means ± SEM (n = 8 mice / time-point and genotype).

**
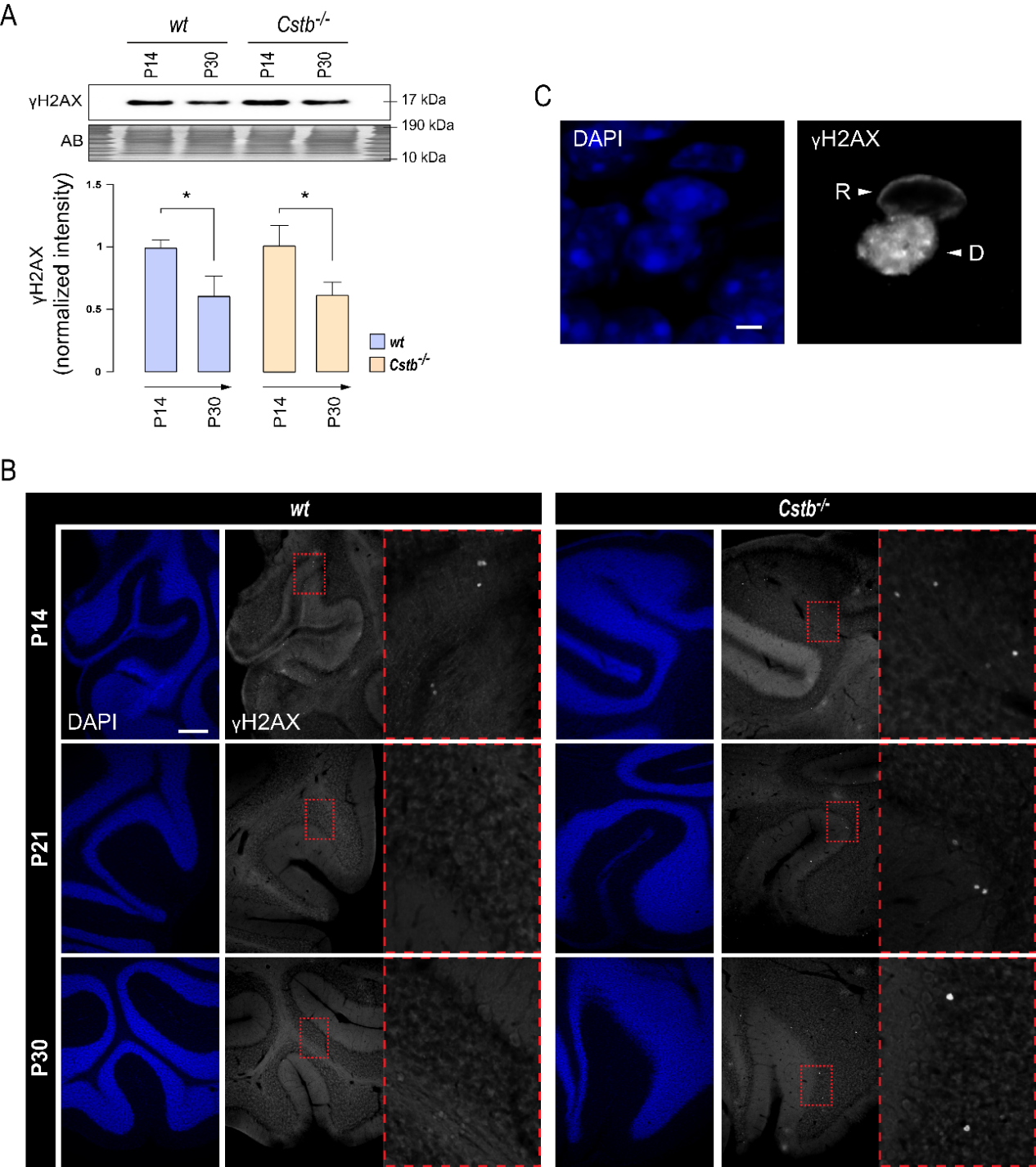
**

**Supplementary Figure 4.** CSTB- and age-dependent changes in γH2AX immunoreactivity during cerebellar development. **(A)** Western blot detection of γH2AX in cerebellar lysates of wt and Cstb-/- mice at P14 and P30. The bar chart illustrates age-dependent changes in γH2AX amount independent of the genotype. Normalized intensity values are plotted as means ± SEM (n = 4-6 mice / time-point and genotype). **(B)** Representative epifluorescence microscopy images of γH2AX immunohistochemical staining in cerebellum of wt and Cstb-/- mice. A scattered cell population presenting a pan-nuclear γH2AX immunoreactivity was consistently observed in the wt cerebellum at P14, and in the Cstb-/- cerebellum at all three time-points analysed. n = 5 mice / time-point and genotype. Scale bar = 200 μm. **(C)** Confocal microscopy image of a P30 Cstb-/- cerebellum stained for γH2AX. White arrowheads show a cell with a diffuse pan-nuclear γH2AX immunoreactivity **(D)** and a cell with an apoptotic-ring-like pan-nuclear γH2AX immunoreactivity (R). Scale bar = 2 μm. * P < 0.05.
